# Spatiotemporal deformation patterns of the Lake Urmia Causeway as characterized by multisensor InSAR analysis

**DOI:** 10.1038/s41598-018-23650-6

**Published:** 2018-04-03

**Authors:** Sadra Karimzadeh, Masashi Matsuoka, Fumitaka Ogushi

**Affiliations:** 1 Department of Architecture and Building Engineering, School of Environment and Society, Tokyo Institute of Technology, 4259-G3-2 Nagatsuta, Yokohama, Kanagawa 226-8502 Japan; 2 Department of GIS and Remote Sensing, University of Tabriz, Tabriz, 5166616471 Iran

## Abstract

We present deformation patterns in the Lake Urmia Causeway (LUC) in NW Iran based on data collected from four SAR sensors in the form of interferometric synthetic aperture radar (InSAR) time series. Sixty-eight images from Envisat (2004–2008), ALOS-1 (2006–2010), TerraSAR-X (2012–2013) and Sentinel-1 (2015–2017) were acquired, and 227 filtered interferograms were generated using the small baseline subset (SBAS) technique. The rate of line-of-sight (LOS) subsidence of the LUC peaked at 90 mm/year between 2012 and 2013, mainly due to the loss of most of the water in Lake Urmia. Principal component analysis (PCA) was conducted on 200 randomly selected time series of the LUC, and the results are presented in the form of the three major components. The InSAR scores obtained from the PCA were used in a hydro-thermal model to investigate the dynamics of consolidation settlement along the LUC based on detrended water level and temperature data. The results can be used to establish a geodetic network around the LUC to identify more detailed deformation patterns and to help plan future efforts to reduce the possible costs of damage.

## Introduction

**Figure 1:**
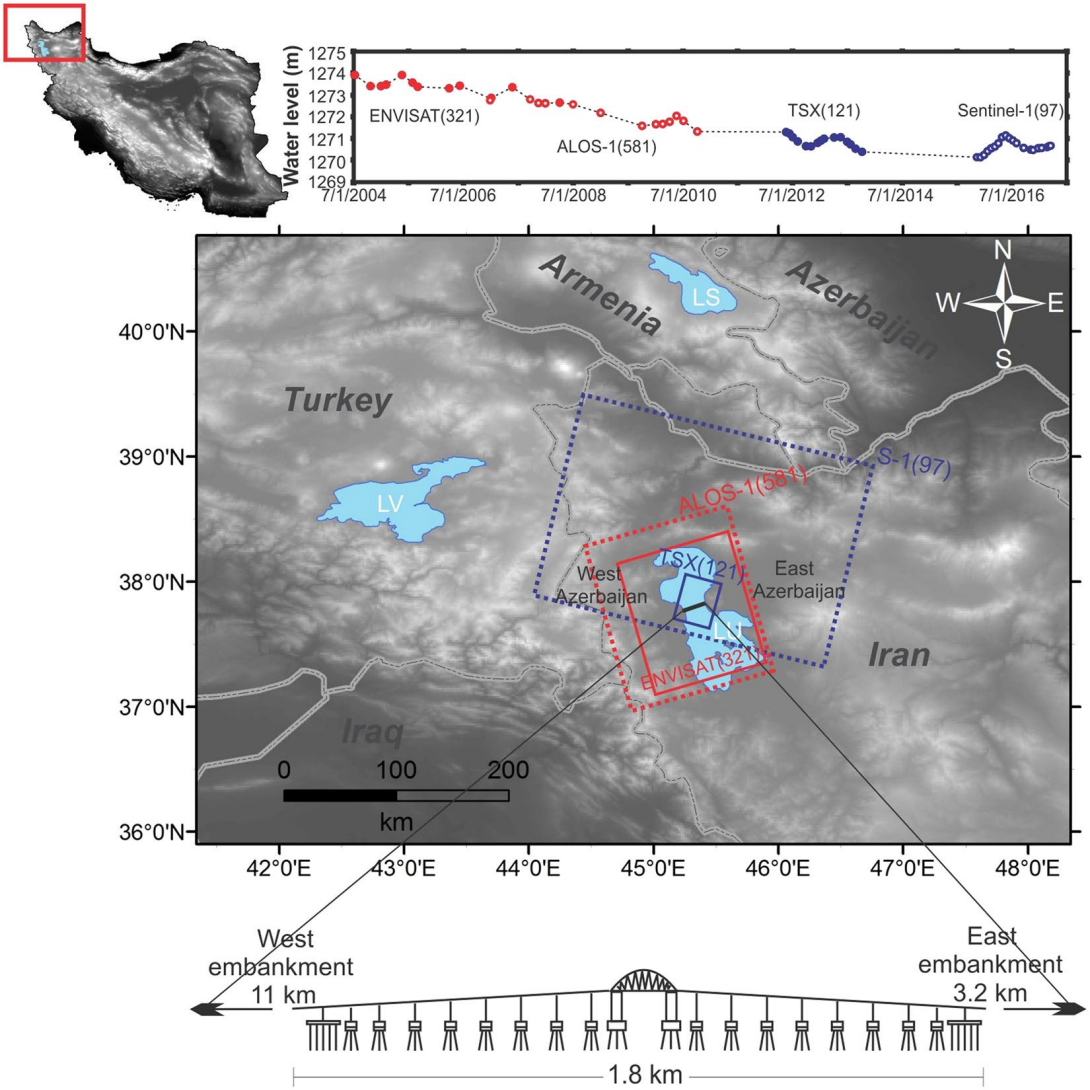
Study area showing the LUC, Lake Urmia (LU), Lake Van (LV) and Lake Sevan (LS). Red and blue rectangles indicate ascending (ALOS-1 and Envisat) and descending (Sentinel-1 and TSX) SAR datasets, respectively. The inset map shows the topography of Iran, which was produced from the 3 arc-second SRTM DEM. The upper graph shows the time series of water level changes and the dates that the SAR datasets were acquired between 2004 and 2017. The nonstationary time series with a semi-seasonal trend shows 3 m of water decline over a period of 13 years. Figures were created in ArcGIS 10.2 (http://www.esri.com/software/arcgis).

From ancient to contemporary times, roads and bridges have played strategic roles in allowing passage over topographic obstacles (e.g., valleys and lakes), saving time and energy and facilitating cultural connections between nations and economic ties between regions^1^. The Lake Urmia Causeway (LUC) is a combination of roads and bridges over Lake Urmia in NW Iran with a total length of approximately 16 km. Lake Urmia, which is the largest saline lake in the Middle East, has a semi-rectangular shape with a length and width of 135 km and 70 km, respectively (Fig. 1). Construction of the LUC over the lake occurred over approximately 25 years. The LUC is composed of three major segments. The west embankment, which is approximately 11 km long, represents the initial phase of construction; it dates back to the early 1980s and connected the narrowest locations of the western and eastern parts of the lake. The east embankment, with an approximate length of 3 km, was constructed in the second phase. A multi-span arch bridge that is approximately 1.8 km long was completed in 2006 after the LUC project was abandoned for many years^2^. Although the LUC has facilitated the transportation of goods and people between the West and East Azerbaijan provinces of Iran, concerns about the impact of dike-type causeways on the water flow and health conditions of Lake Urmia have increased^2,3^. However, AghaKouchak *et al*. hypothesized that Lake Urmia has fallen victim to the Aral Sea syndrome, or lake desiccation that arises primarily from the mismanagement of water reservoirs, intensive development of agricultural and anthropogenic activities and upstream competition to control water^4^. The descriptive map of the study area in Fig. 1 shows details of the bridge and the 3 m water decline (from 1274 m to 1271 m) of the lake in conjunction with the dates of acquired synthetic aperture radar (SAR) datasets between 2004 and 2017. The bridge was constructed over 18 caissons and two main concrete collars at the beginning and end of the bridge. These caissons and collars are connected to the lake bed using vertical and oblique steel piles with depths of over 80 m. The bridge carries two single-direction main roads (each 9.5 m wide) and a railway (5 m wide) for a total width of 24 m. The maximum width and height of the bridge are 28 m and 12 m above sea level, respectively^5^.

Interferometric synthetic aperture radar (InSAR) is an effective technique that exploits the phase difference (i.e., differential interferogram) between SAR images of the same area with the same imaging direction and geometry, which are obtained at different times. Multi-temporal InSAR techniques can be used to map surface and structural deformations with acceptable accuracies in both temporal and spatial scales, which allows abnormal movements and potential challenges of civil projects over time to be analysed using the associated time series^6–14^. We present a comprehensive multisensor deformation analysis of the LUC using InSAR, which reveals the natural settlement rates of the east embankment and the artificial uplift rates at the beginning of the west embankment. The rate of settlement accelerated between 2012 and 2013, whereas uplift was observed only between 2013 and 2016 and was related primarily to road extensions and minor earthwork. We used InSAR small baseline subset (SBAS) techniques on several SAR satellite datasets, from both ascending and descending orbits, to provide multiple time series across the LUC and to demonstrate the concept of structural SAR monitoring via principal component analysis (PCA)^13,14^.

## SAR Data Description

We use 12 C-band Envisat ASAR images (orbit 321) from 7 July 2004 to 2 April 2008, 13 L-band ALOS-1 FBS/FBD images (orbit 581) from 26 December 2006 to 6 October 2010, 15 X-band TerraSAR-X (TSX) images (orbit 121) from 20 May 2012 to 8 October 2013 and 28 Interferometric Wide Swath (IW) C-band Sentinel-1 (orbit 97) images from 11 November 2015 to 3 July 2017 in the SBAS analysis. Note that the Envisat and TSX datasets contain single-polarization data in VV and HH polarizations, respectively. The ALOS-1 dataset is a mixture of both single (HH) and dual (HH and HV) polarizations, whereas the Sentinel-1 data are all dual-polarization data in VV and HV modes (Supplementary Table 1). To ensure consistency in the SBAS analysis, cross-polarization datasets are neglected, and only VV and HH polarizations are considered. In total, 25 ascending and 43 descending single look complex (SLC) images were used to produce 227 differential interferograms under pre-defined conditions. For the Envisat and ALOS-1 datasets (which had relatively large revisit intervals), we assigned a temporal baseline of less than 730 days, whereas for the TSX and Sentinel-1 datasets, we used a threshold of 180 days due to the shorter gap between the acquired images. In the Envisat and ALOS-1 datasets, we used all of the spatial baseline possibilities due to the sparse number of images in the relative timespans, but the spatial baselines of the TSX and Sentinel-1 datasets were limited by up to 40% of the relative maximum normal baseline. The maximum relative baselines for Envisat, ALOS-1, TSX and Sentinel-1 were approximately 1800 m, 3850 m, 480 m and 200 m, respectively. The SAR footprints and the arrangement of the interferograms of the Envisat, ALOS-1, TSX and Sentinel-1 datasets are shown in Fig. 1 and Supplementary Fig. 1. The use of pre-defined temporal and spatial conditions helps avoid decorrelations. To decrease the level of speckle noise in the SLC images and increase the reliability of the coherence maps, we set the range and azimuth factors for each dataset. The pixel spacing in the range and azimuth directions for all of the data was approximately 20 × 20 m, but the presented multi-look factors sometimes result in rectangular pixels. For example, we could set the range to 6 and the azimuth to 1 for the Sentinel-1 data, but the pixels become rectangular (22 × 13 m), which made the interpretation difficult. Because the grid size is set to tune the range and azimuth looks, the closest spatial resolution to the 20 × 20 m grid was selected for the convenience of the interpretation. Thus, we set the range and azimuth factors to 4 and 1, respectively, for the multi-look Envisat images, 2 and 4, respectively, for the multi-look ALOS-1 images, 9 and 10, respectively, for the multi-look TSX images, and 5 and 1, respectively, for the multi-look Sentinel-1 images. For each dataset, single images were taken as reference images (red circles in Supplementary Fig. 1a–d); all of the processed slant range pairs were co-registered according to the geometric parameters of the relative reference image. The reference images were then selected based on how well distributed they were in the SBAS network while maximizing the number of connections to other images. The mean coherence value of the SBAS networks in Supplementary Fig. 1 shows that the quality of the produced interferograms in the later datasets is better due to small temporal or spatial baselines. (Note, however, that the earlier datasets are still satisfactory.) The numbers of coherence pairs in the Envisat, ALOS-1, TSX and Sentinel-1 datasets are 59, 49, 52 and 67, respectively, and the relative mean spatial baselines for Envisat, ALOS-1, TSX and Sentinel-1 are 452 m, 1449 m, 126 m, and 55 m, respectively. The large spatial baselines in the ALOS-1 datasets are related to an altitude change during ALOS-1 operation between 2007 and 2008 to obtain satisfactory data^15^.

## Results

### InSAR Patterns and Field Survey

**Figure 2:**
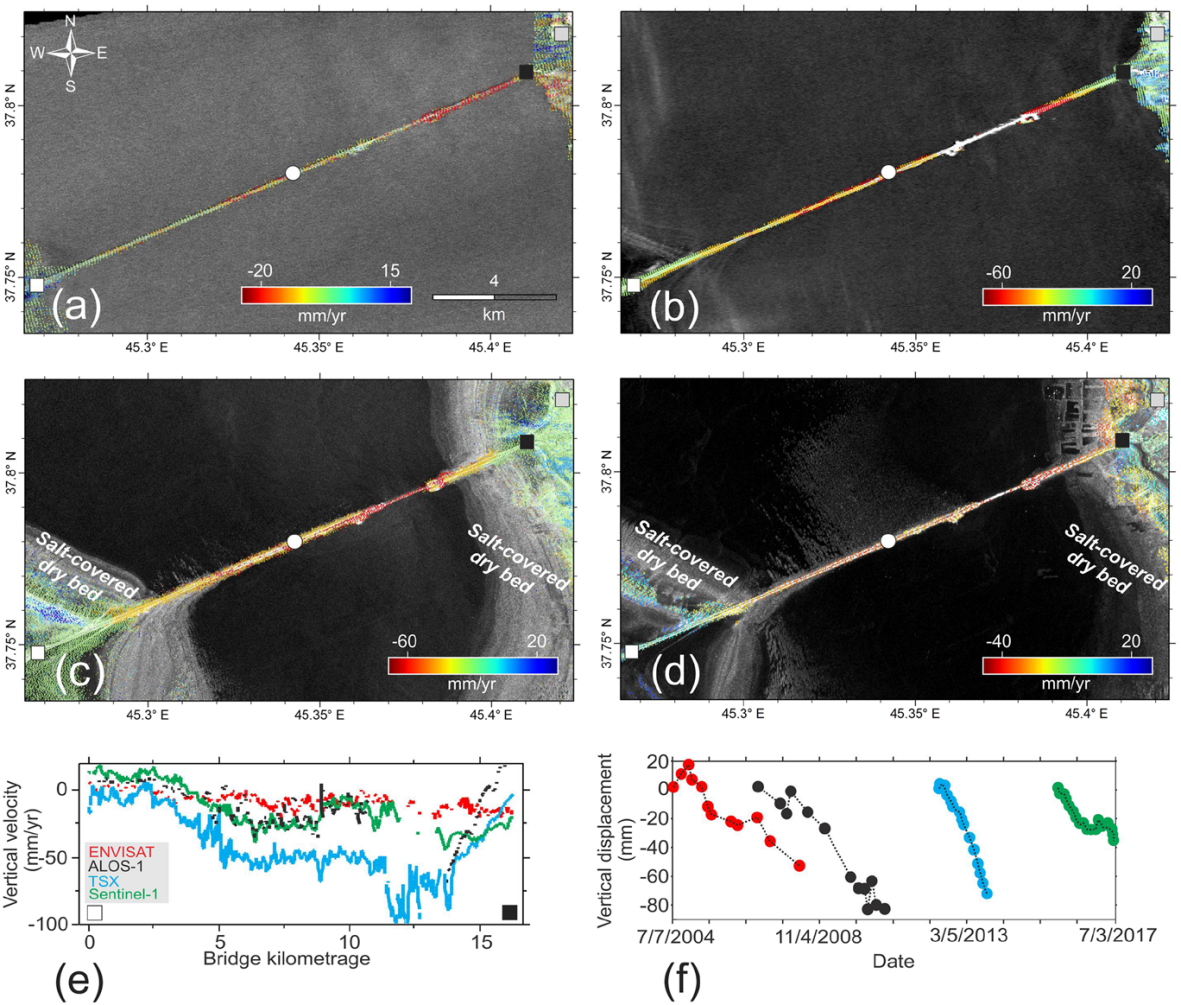
InSAR vertical velocity/displacement of the LUC in Iran. (**a**) Envisat ascending velocity map covering July 2004–April 2008. (**b**) ALOS-1 ascending velocity map covering December 2006–October 2010. (**c**) TSX descending velocity map covering May 2012–October 2013. (**d**) Sentinel-1 descending velocity map covering November 2015–July 2017. (**e**) Profiles drawn across the deformation maps with widths of 50 m from west (white square) to east (black square). (**f**) Time series fluctuations of an arbitrary point (white circle) in the middle of the LUC. The SBAS time series was generated using SARMAP’s SARscape package 5.4 (http://www.sarmap.ch/), and ArcGIS package 10.2 (http://www.esri.com/software/arcgis) was used to create the figures.

First, we note that the SBAS deformation velocities are relatively insensitive to north-south movements due to the inherent nature of polar-orbit SAR satellites. Thus, the line-of-sight (LOS) patterns are converted to east-west and vertical patterns. In the absence of auxiliary SAR data from ascending and descending orbits of the used datasets and the lack of GPS measurements in the study area, the deformation values in the vertical and east-west directions can be calculated as follows:

1 $${D}_{LOS}={D}_{V}cos\theta -{D}_{EW}sin\theta cos\alpha $$

where *D*_*LOS*_ is the deformation in the LOS of the satellite, *θ* and *α* are the incidence angle and azimuth of the LOS, and *D*_*V*_ and *D*_*EW*_ are the deformations in the vertical and east-west directions, respectively^16^. We then assumed that the east-west movement is insignificant and, because of hydrostatic loads, we expect the deformation to occur in only the vertical direction (Fig. 2a–d**)**. All of the InSAR velocities are calculated with respect to an area of zero deformation (reference point) in the study area. The zero deformation area for the relative velocity maps was selected by the following criteria: 1- north of the LUC, far from the deformation; 2- based on high coherence values in the produced interferograms. In earlier missions (Fig. 2a,b), the rate of long-term subsidence in the east embankment of the LUC between 2004 and 2010 was higher than that in the west embankment, whereas in later missions (Fig. 2c,d), the deformation patterns were more uniform across the LUC. The peak subsidence observed in the TSX dataset occurred between 2012 and 2013 and was as high as 90 mm/yr. As shown in Fig. 2a–d, after 2012, salt-covered dry beds emerged to the east and west of the LUC. In addition, the water level of the lake was at its lowest level, which indicates that the majority of the downward movements of the LUC resulted from long-term consolidation, by which the soils beneath the embankment decreased in volume. The subsidence rates obtained from the Envisat dataset for 2004–2008 and the ALOS-1 dataset for 2006–2010 did not exceed 40 mm/yr and 70 mm/yr, respectively. The SBAS rates of Sentinel-1 (Fig. 2d) from 2015 to 2017 indicate that the rate of subsidence has decreased in recent years due to an increase in the water level of the lake but that subsidence continues. At the western entrance of the LUC, the rate of uplift based on the Sentinel-1 data is as high as 15 mm/yr, which is probably due to road construction. Figure 2e shows a profile of each dataset along the LUC. Some uplift in the green profile (related to Sentinel-1) occurred between 0 and 3 km, and the major subsidence in the blue profile (TSX) occurred between 3 and 13 km. The SBAS profiles of the Envisat and ALOS-1 datasets (red and black lines, respectively) are smoother, but the ALOS-1 data indicate a sudden uplift in the east embankment^17^. In addition, the time series fluctuations of an arbitrary point in the middle of the LUC (Fig. 2f) show that the rate of subsidence was higher between 2012 and 2013 than during other periods.

**Figure 3:**
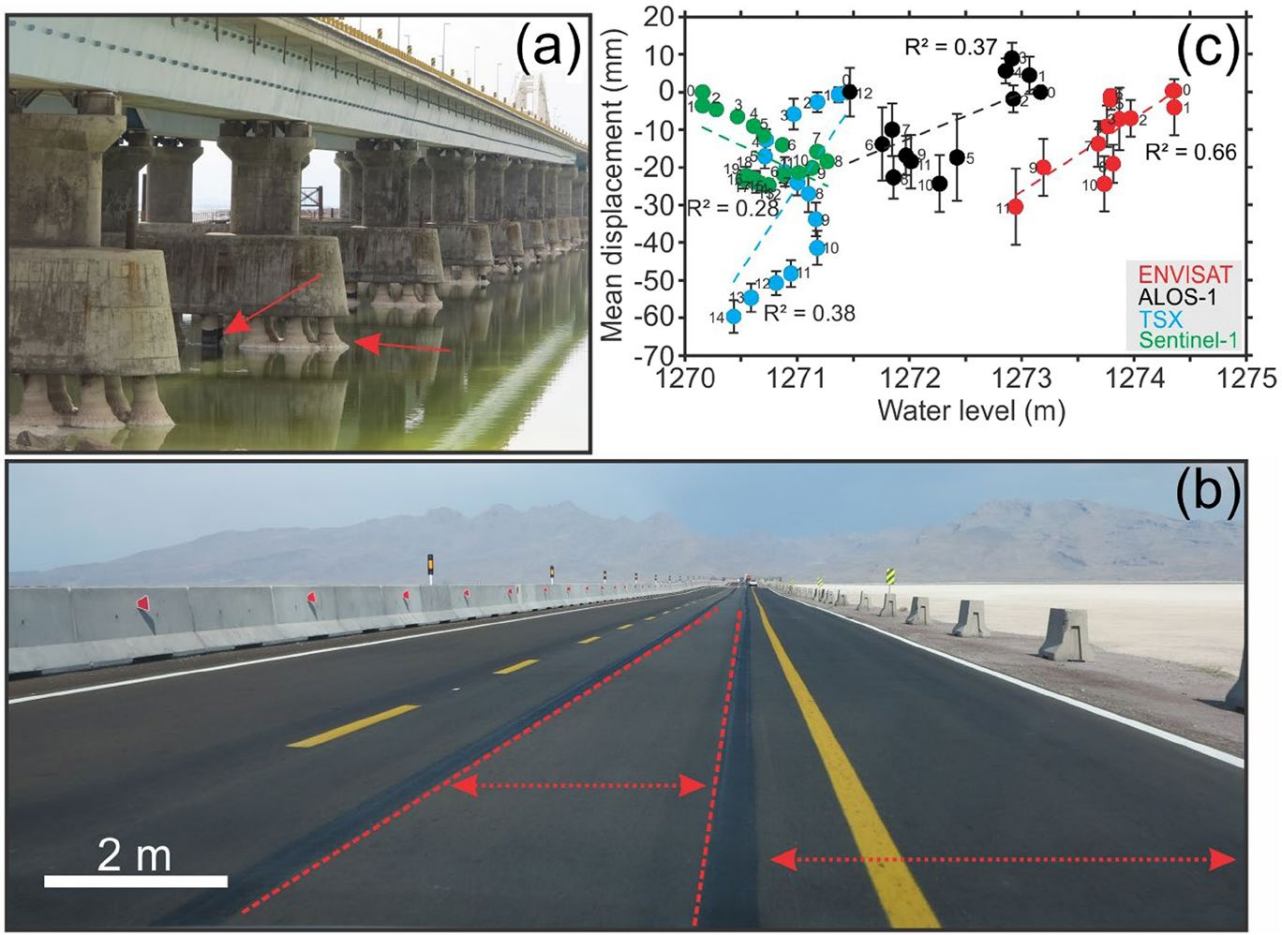
Field photographs and data. (**a**) Columns and spans of the bridge. The red arrows indicate salt-plated columns and rusted steel columns. (**b**) Road expansion operations in the west embankment during recent years. (**c**) Mean vertical displacement of each dataset versus the mean water level of the lake. The coloured dashed lines indicate linear regressions of each dataset. The error bars show the standard deviations of 200 randomly selected time series for each date. The numbers associated with the dots indicate the temporal sequences of the SBAS analysis in Supplementary Fig. 1.

We conducted a field visit during the summer of 2017 to observe the physical conditions of the LUC and its relative deformations. Figure 3a shows the multispan arch bridge, where water can circulate between the regions of the lake north and south of the bridge. Due to the high salinity of the lake, most of the columns are salt-plated, and heavy rusting of the steel columns has occurred despite technical treatments (Fig. 3a). As discussed previously, significant vertical deformations are inferred from recent TSX and Sentinel-1 data. Because of the data availability and finer data resolution, we were able to detect both long-term subsidence and transient deformation of the LUC and identify traces of recent deformation during the field observations. We found that the observed transient deformation (uplift) detected by Sentinel-1 was associated with subgrade preparation and road-expansion projects. As shown in Fig. 3b, the locations of the lanes and line markings have changed, and the width of the road has increased approximately 4 m.

To investigate the possible relationships between the water level and road settlement due to soil consolidation, we also obtained the daily water levels of the lake for the same dates as the SAR acquisitions. Except for 8 dates from the Sentinel-1 dataset (between March 2017 and June 2017), the water levels of all of the acquisitions and their corresponding vertical displacements are available. Figure 3c shows a comparison of the displacements and water level changes. If the records are contaminated by seasonal and anthropogenic changes, the coefficient of determination (*R*^2^) would be a better indicator of the relationship between the water level and displacement than the correlation coefficient (*R*). The calculated *R*^2^ values for the Envisat, ALOS-1, TSX and Sentinel-1 datasets are 0.66, 0.37, 0.38 and 0.28, respectively. The *R*^2^ value is low in all the datasets except in the Envisat dataset, possibly because the water record station is not located exactly under the LUC. The low *R*^2^ value between the Sentinel-1 displacements and the water levels manifests itself more than in the other datasets, indicating that the regression line does not perfectly fit the data, possibly due to seasonal changes and anthropogenic activity after 2015, which was when the Urmia Lake Restoration Program (ULRP) was established to save the lake via extensive monitoring of water usage and stopping upstream development projects. In the next subsections, we present results of PCA and a hydro-thermal model to interpret the deformation along the LUC using detrended water level and temperature data together with the Sentinel-1 InSAR displacements.

### PCA Patterns

**Figure 4:**
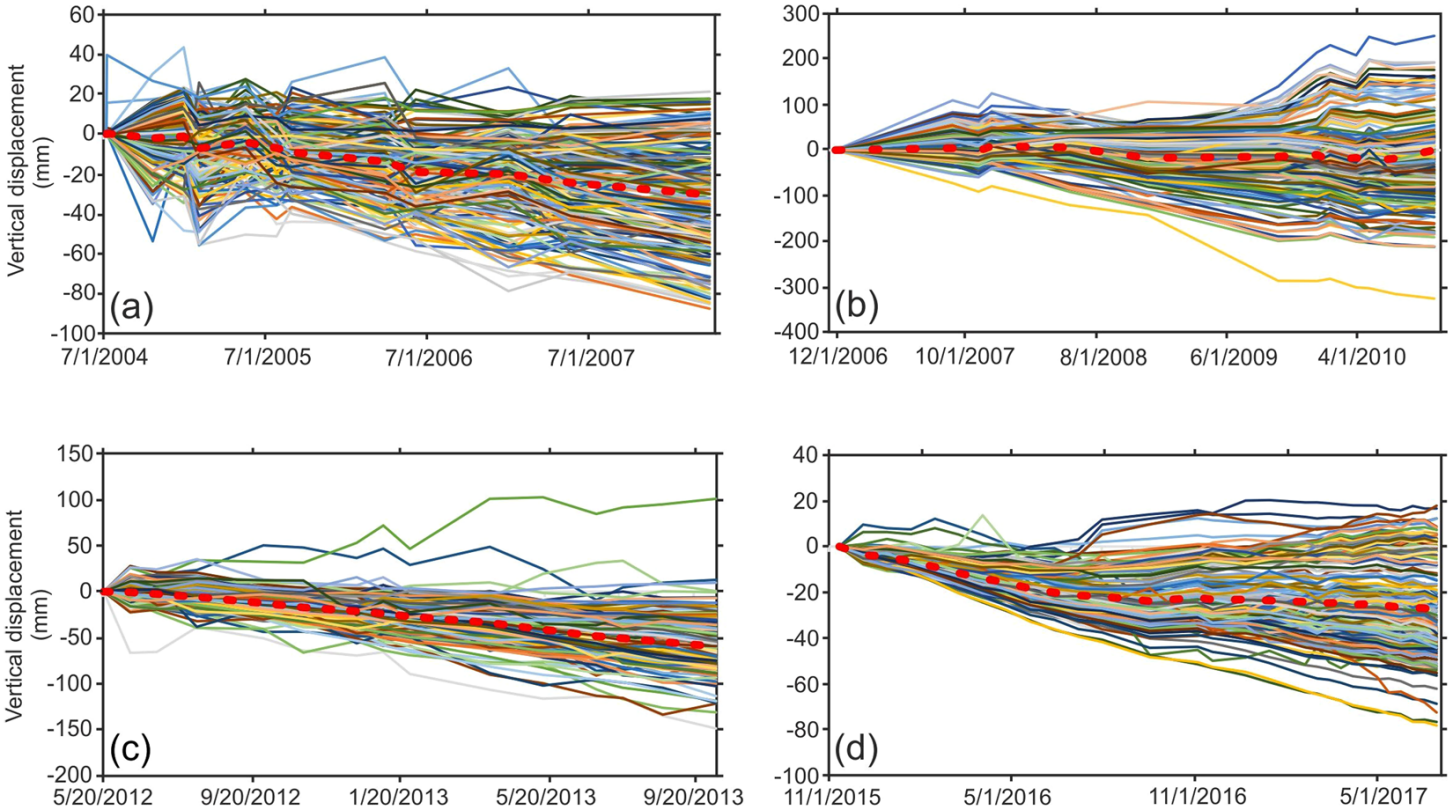
Time series of 200 samples from (**a**) Envisat, (**b**) ALOS-1, (**c**) TSX and (**d**) Sentinel-1. The red dashed lines represent the mean displacements.

Several previous studies^17–19^ have used finite element modelling (FEM) to investigate the long-term road subsidence of the LUC. As an alternative, we consider the LUC as a multivariate process via a PCA model. The main reason we assume that the LUC is an integrated road is that if one part is damaged, the entire structure loses its functionality. We obtained thousands of time series of data from the LUC and from independent SBAS analyses for their specific timescales. To consistently analyse the datasets from the four sensors, we randomly chose 200 time series across the bridge (Fig. 4). Seasonality is not detected in most of the selected samples. As shown in Fig. 4, except for the mean values of the time series (red dashed line), which show gradual subsidence over time, the space bounded by the entire time series is too wide to detect important trends associated with the LUC. PCA uses multiple dimensions (here, displacement time series) and flattens them into 2 or 3 dimensions to reveal new comparative information.

**Figure 5:**
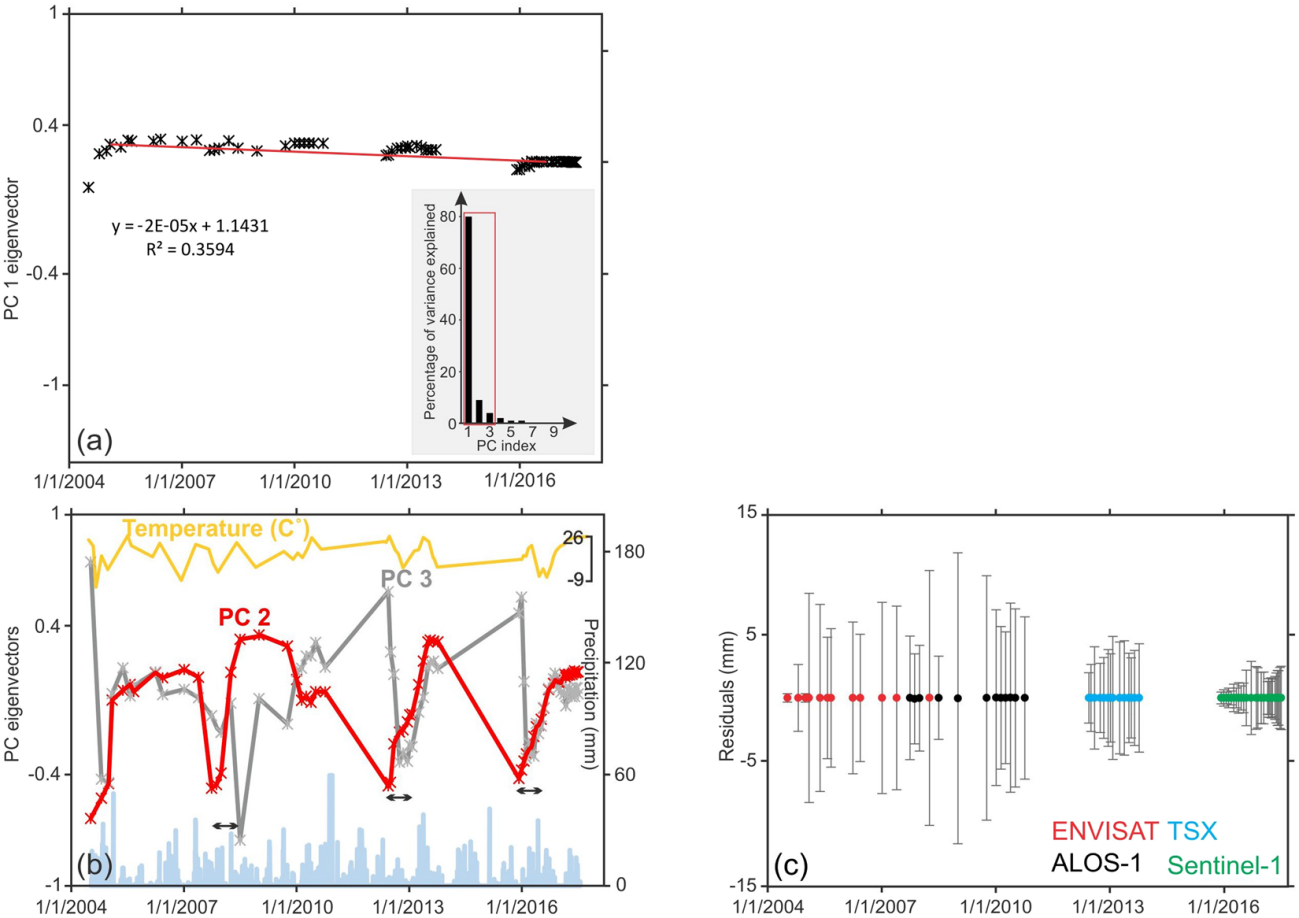
Results of the PCA. (**a**) Loadings of PC1 together with the scree plot of the percentage of variance explained by each PC. (**b**) Loadings of PC2 and PC3 together with the precipitation rate (blue bars) and temperature (yellow line) measured at a meteorological station 30 km from the LUC. The black double arrows denote the time lags between the selected troughs of PC2 and PC3. (**c**) Residuals and standard deviations of the variables for each dataset.

To extract the principal components (PCs) before applying eq. (9), every individual time series must first be centred. We subtract the average value from all 200 observations for which the average number of observations would be zero after the transformation (Supplementary Fig. 4). Each of the three PCs corresponds to a score and a loading that best describes the shape of the variations (see Supplementary Information and figures). The PCA results show that PC1 explains 80% of the variance, and PC2 and PC3 explain, respectively, 9% and 4% (Fig. 5a). Thus, the first three components of these data can represent almost the entire dataset. To ensure that the PCs are consistent with the general behaviours of the LUC, we performed augmented Dickey-Fuller (ADF) test. The ADF test is similar to Dickey-Fuller (DF) test, but it can handle more complex models. If we do not apply unit root tests such as the ADF test, which is a common regression test of time series, our results can potentially be meaningless. We used the PCs from the datasets and performed an ADF test to capture the stable conditional means over time. The ADF test is based on the following three assumptions: (1) the PC does not have a zero mean (no constant), (2) the PC does not have a non-zero mean (constant only), and (3) the PC has a trend but does not have a non-zero mean.

2 $${\rm{\Delta }}{Y}_{t}=\gamma {Y}_{t-1}+\sum _{j=1}^{p}({\delta }_{j}{\rm{\Delta }}{Y}_{t-j})+{e}_{t},$$

3 $${\rm{\Delta }}{Y}_{t}=\alpha +\gamma {Y}_{t-1}+\sum _{j=1}^{p}({\delta }_{j}{\rm{\Delta }}{Y}_{t-j})+{e}_{t},$$

and

4 $${\rm{\Delta }}{Y}_{t}=\alpha +\beta t+\gamma {Y}_{t-1}+\sum _{j=1}^{p}({\delta }_{j}{\rm{\Delta }}{Y}_{t-j})+{e}_{t},$$

where *Y* is an observed time series, *t* is the time index, *α* is a constant (drift), *β* is the coefficient of a time trend, *γ* is the coefficient presenting the process root, *e*_*t*_ is an independent residual term, and *p* is the lag order of the first-differences autoregressive process. The ADF test determines whether the criteria are met and returns a “TRUE” statement (if the calculated probability based on the null hypothesis is smaller than the probability threshold) or “FALSE” statement (if the calculated probability based on the null hypothesis is larger than the probability threshold) when the PCs are stationary or non-stationary, respectively. The results of the stationary test show that PC1 is “TRUE” or stationary under the second and third assumptions. Thus, we can regard PC1 as the long-term component of the LUC. As shown in Fig. 5, PC1 in all datasets seems to be constant, which means that a long-term downward trend is present. In the Envisat data, PC2 and PC3 are “TRUE” or stationary under the second and third assumptions. In the ALOS-1 dataset, only PC2 under the second assumption results in a stationary time series. In the TSX and Sentinel-1 datasets, PC3 is stationary, whereas only PC2 is stationary in the Sentinel-1 dataset under the second and third assumptions (Supplementary Table 2). PC2 and PC3 are relatively complex and behave differently from PC1. We assume that if the results of the ADF test are “FALSE” for at least one of the above-mentioned models, they are likely associated with seasonal effects, and we can proceed with a simple seasonality test that compares the period variance with the residual variance of the PCs. The seasonality test of PC1 was negative, but the test indicated the presence of seasonal effects in PC2 and PC3, as shown by their eigenvector time series in Fig. 5b. The peaks and troughs in PC2 and PC3 match the precipitation rate (blue bars in Fig. 5b) in only a few cases, potentially due to the 30 km distance between the precipitation recording station and the LUC and irregular precipitation due to the semi-arid climate in NW Iran. Troughs in PC3 in the summers of 2008 and 2012 and winter of 2016 occur a few months later than those for PC2 (double arrows in Fig. 5b). This result suggests that PC3 is likely related to the second phase of deformation, i.e., delayed residual deformation. In this study, temperature records can better describe the seasonal behaviours of PC2 and PC3 than can the precipitation rate. A comparison of temperature records (yellow line in Fig. 5b) and two seasonal components indicates that the seasonal deformation described by PC3 is likely related to the thermal expansion and contraction of the LUC during hot summers and cold winters. Moreover, the mean residuals of the PCA model and the samples in Fig. 5c indicate that the model is well represented by all of the datasets; however, the standard deviations in the latter TSX and Sentinel-1 datasets are considerably smaller, which is related to their highly repeated observations and the lower uncertainty of the SBAS-based displacement calculation. The small standard deviations also imply that homogenous displacement with less influence of seasonal factors on the LUC has continued in recent years.

### Time-displacement modelling

The Hydrostatic-Seasonal-Time (HST) and Hydrostatic-Temperature-Time (HTT) models, which are statistical deterministic models that are specifically designed for health monitoring of concrete structures, such as dams and causeways, based on the interpretation of the measured displacements from direct or inverted pendulums, can be used to understand how the LUC responds to stress^12^. The model we apply here to investigate non-linear deformations along the LUC is composed of the elapsed time, temperature and water level. Thus, a time series of the temperature from 1 January 2004 to 15 August 2017 is obtained from the closest station to the LUC (Supplementary Fig. 2). Because of the different incidence angles of the Envisat, ALOS-1, TSX and Sentinel-1 datasets, the lack of dense SAR observations in earlier missions and the lack of water level time series in some epochs, re-projecting all four datasets into the same geometry radial is difficult. Therefore, we apply only the HTT model to the PCA scores of the Sentinel-1 results from 11 November 2015 to 17 March 2017. The HTT model is composed of a polynomial function with linear (thermal and irreversible effects) and exponential (description of hydraulic load by water elevation) portions. According to Milillo *et al*., the displacements can be calculated as follows^12^:

5 $${D}_{HTT}=aT+b+c{H}_{w}+d{H}_{w}^{2}+e{H}_{w}^{3}+f\,{H}_{w}^{4}+gt$$

where *D* is the displacement (mm), *T* is the temperature (°C), *H*_*w*_ is the water elevation (m), *t* is the elapsed days from 11 November 2015 to 17 March 2017, *a* − *f* are coefficients estimated using least squares and iteration methods, and *g* is the ageing parameter.

**Figure 6:**
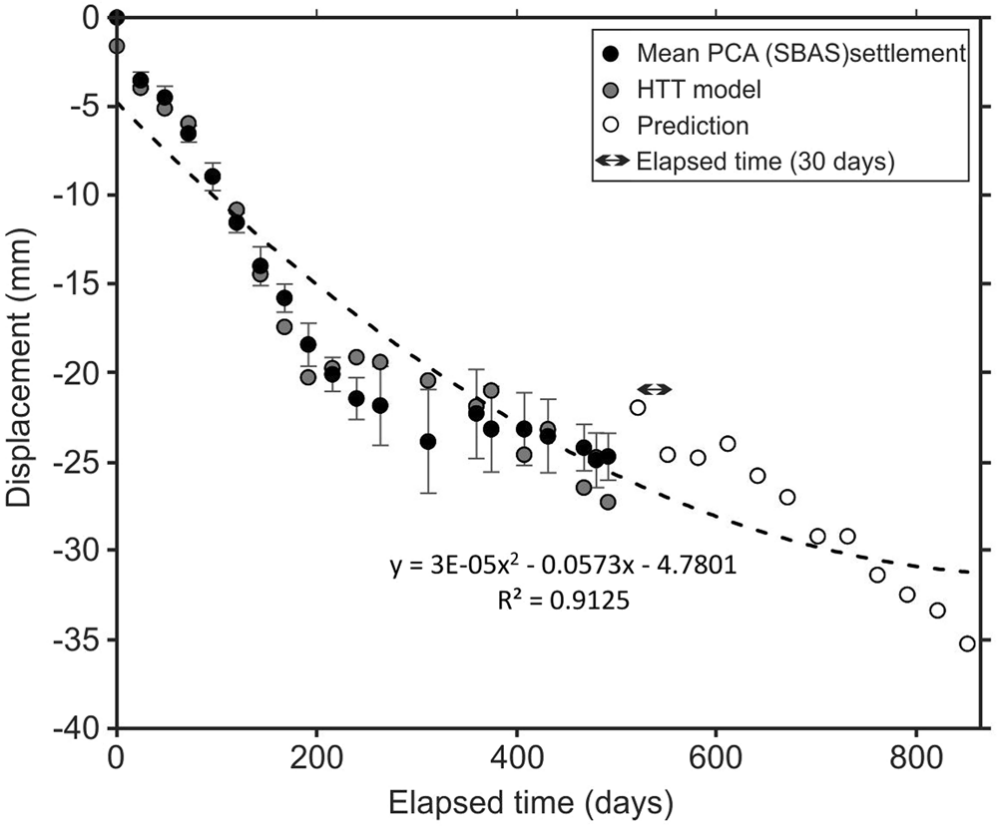
Mean displacements of the Sentinel-1 dataset from the PCA (black circles), modelled displacements (grey circles) and the 1 year of predicted displacements (white circles).

Both the temperature and water level data show periodic behaviours, but the water data display a downward trend (Supplementary Fig. 2) that must be detrended. The detrending consists of removing the effects of anthropogenic activity from the trend to allow only the absolute changes to be identified. It can be beneficial to identify and remove trend information before time series modelling. Since the water level time series shows a downward trend, to perform a fair hydro-thermal analysis, we detrended all the datasets and estimated the thermal-hydrological component and consolidation term along the LUC for the vertical displacements. The results for a time span of 492 days show how repeated InSAR observations can characterize the dynamic behaviour of the LUC. Table 1 shows the estimated parameters of the HTT model along the LUC. One of the parameters that is not correlated with the thermal and hydrological terms is the “ageing term” or irreversible displacement, which is considered an indicator of the potential consolidation rate of the LUC. It has a small value because consolidation can sometimes take several years. The water takes an extremely long time to drain out of the soil because the hydraulic conductivity of saturated clays is very low. Since the time for consolidation is predictable, we predicted the displacements for 365 days in the future. Figure 6 shows the displacements from the PCA results, the modelled displacements and the 1 year of predicted displacements. Assuming an ageing coefficient of −0.04 mm/day, the cumulative consolidation settlement for the next 365 days is approximately −14 mm, and the second-order trend line of the data and predictions shows that it decreases with time.

**Table 1:** Output parameters of the polynomial function for the HTT model.

Description		HTT model (eq. 5)
Thermal	a	0.17 mm/°C
	b	0.0001
Hydrostatic	c	0.1
	d	0.15
Pressure	e	−0.002
	f	−3.9E-05
Ageing	g	−0.04 mm/day
RMSE	1.6

## Discussion and Conclusions

Although the Envisat and ALOS-1 results provide reliable data that allow the identification of simple early patterns of deformation, many ambiguities remain that could not be explored due to the passage of time and the lack of a sufficient number of observations. For example, some human activities on the east embankment were observed by ALOS-1 between 2009 and 2010, but we cannot completely confirm those activities via field investigations because of the time that has elapsed. In contrast, repeated TSX and Sentinel-1 observations over shorter periods revealed both road subsidence patterns and transient artificial changes at the beginning of the west embankment, which can be confirmed through field inspections. The highest rates of road subsidence for the Envisat, ALOS-1, TSX and Sentinel-1 data obtained over their respective measurement periods were approximately 40 mm/yr, 70 mm/yr, 90 mm/yr and 60 mm/yr, respectively.

The mean value of 200 randomly selected SBAS time series indicates that the current behaviour of the LUC involves minor linear consolidation. In the absence of continuous ground-based geodetic measurements, PCA can provide versatile inversion models capable of accommodating multiple datasets over multiple timescales. PC1 corresponded to the long-term lowering of the LUC, whereas PC2 and PC3 highlighted seasonal deformations. However, the behaviours of PC2 and PC3 did not correlate with the precipitation rate, possibly due to the arid to semi-arid climate of the study area and the great distance between the climate station and the LUC. Moreover, the sub-sampled time series reduced the quality of the PCA results and made the interpretation difficult. Further analysis, such as independent component analysis (ICA), could be conducted in the future to differentiate noise in the entire SBAS time series and deformation from multiple independent sources that simultaneously occur. Although the difference between the PCA model and the actual displacements is small, the resulting standard deviation of the ALOS-1 datasets is much higher than those of the other datasets. This finding is also related to the non-uniform settlements in the east and west embankments, the insufficient observations and the insensitivity of L-band data for large deformations due to the long wavelength^16^. The wavelength of the ALOS-1 L-band is approximately 24 cm, and the sensitivity of the displacement is half of the wavelength. Therefore, the C-band (~6 cm) and X-band (~3 cm) are considered to be able to detect smaller displacements than the L-band.

The hydro-thermal model based on the Sentinel-1 PCA scores provided descriptive information about the LUC and indicated that the subsidence was caused by soil consolidation. When water is removed from soil, the pore water pressure increases because the soil carries part of the applied stress. However, validation of the model was difficult due to the lack of ground-based geodetic observations, and because the temperature data were obtained 30 km away from the LUC, our model was not optimal. By applying adjustable thresholds for the parameters and defining the soil properties of different layers, the model may produce more accurate results.

Because damage to one part of the LUC can affect the entire lake’s functionality, we conclude that the contributions of the retired and new SAR missions can help to better evaluate the long-term consolidation and transient deformations. If the settlement that has occurred since 2004 continues into the future, the LUC might be damaged by uneven settlement rates in the east and west embankments. Thus, multisensor InSAR results are expected to be valuable for establishing a geodetic network around the LUC to identify more detailed deformation patterns and to help plan future efforts to reduce the possible costs of damage.

## SBAS and PCA Methods

In our comprehensive analysis of 13 years of SBAS data, we used the methodology of Berardino *et al*.^20^ to obtain a mean displacement velocity and to analyse the corresponding time series of differential interferograms. We began the SBAS analysis by considering *N* + 1 SAR images over the same geographic region at ordered times (*t*_0_,…,*t*_*N*_) with the assumptions that all of the images are co-registered to a single “master image” and that at least one image can facilitate interferometric analysis with another image. As a result, each SBAS is composed of a minimum of two acquisitions. Accordingly, the number of possible differential interferograms (*M*) is used to estimate a low-pass signal component, which can be defined as follows if we assume that *N* is odd.

6 $$\frac{N+1}{2}\le M\le N(\frac{N+1}{2})$$

The coordinates of each pixel in the range and azimuth directions (*x*,*r*) of the generic unwrapped multi-look *j*-interferogram were considered for the SAR acquisition times *t*_*B*_ and *t*_*A*_ and are provided by

7 $$\begin{array}{ccc}\delta {\phi }_{j}(x,\,r) & = & \phi ({t}_{B},\,x,\,r)-\phi ({t}_{A},\,x,\,r)\approx \frac{4\pi }{\lambda }[{d}^{LP}({t}_{B},\,x,\,r)-{d}^{LP}({t}_{A},\,x,\,r)]\\  &  & +\,{\rm{\Delta }}{\phi }_{j}^{atm}({t}_{B},\,{t}_{A},\,x,\,r)+{\rm{\Delta }}{\phi }_{j}^{topo}(x,\,r),\end{array}$$

where *j* is an integer between 1 and *M*, *φ*(*t*_*B*_, *x*, *r*) and *φ*(*t*_*A*_, *x*, *r*) are the associated multi-look phase components of the two images used to generate the interferogram, *d*^*LP*^(*t*_*B*_, *x*, *r*) and *d*^*LP*^(*t*_*A*_, *x*, *r*) are the LOS deformations of the low-pass components accumulated from *t*_*A*_ to *t*_*B*_ with respect to the instant *t*_0_ as the reference time, *λ* is the wavelength of each satellite, ${\rm{\Delta }}{\phi }_{j}^{atm}({t}_{B},\,{t}_{A},\,x,\,r)$ is the atmospheric phase associated with atmospheric turbulence between two acquisitions, and ${\rm{\Delta }}{\phi }_{j}^{topo}(x,\,r)$ is the topographic phase of the Earth features, which is defined as follows:

8 $${\rm{\Delta }}{\phi }_{j}^{topo}(x,\,r)\approx \frac{4\pi }{\lambda }\frac{{B}_{\perp j}{\rm{\Delta }}z(x,\,r)}{rsin\theta },$$

where *B*_⊥*j*_ is the perpendicular baseline between two acquisitions, *θ* is the incidence angle of the SAR sensors (approximately 23° for Envisat, 39° for ALOS-1, 26° for TSX and 42° for Sentinel-1), and ${\rm{\Delta }}z(x,\,r)$ is a topographic artefact that can be removed or reduced using digital elevation models (DEMs). In eq. (7), the noise effects (which are primarily associated with instrumental performance) are assumed to be negligible. Refinement and re-flattening of the interferograms to remove the turbulent phases were performed by selecting approximately 100 ground control points in motionless and highly coherent areas and using a 1 arc-second Shuttle Radar Topography Mission (SRTM) DEM and a third-order polynomial refinement equation. Equations (7) and (8) describe a system with infinite solutions because of the *N* + 1 unknowns ([*φ*(*t*_1_, *x*, *r*), …, *φ*(*t*_*N*_, *x*, *r*), Δ*z*(*x*, *r*)]). The singular value decomposition (SVD) method, which does not require prior information about the temporal behaviour of the deformed area, provides a simple solution to invert the system. In the final step of refining the surface deformation data (*d*^*LP*^(*t*_*i*_, *x*, *r*); *i* = 0, …, *N*), the phase related to the atmospheric effects $({\rm{\Delta }}{\phi }_{j}^{atm}({t}_{B},\,{t}_{A},\,x,\,r))$ must be detected and mitigated by considering that the possible atmospheric phase components are clustered primarily in space and time^21,22^. Pixels with coherence values smaller than our defined threshold (0.3) were set to dummy variables (NaN), and differential interferograms were unwrapped via a minimum cost flow method^23^. Spatial low-pass and temporal high-pass filtering were then applied to the deformation map to obtain the SBAS deformation.

The output of the SBAS for one dataset is one matrix or one table of data, and we follow the convention that samples (i.e., displacements in different locations) are kept in different rows and that the variables (dates) are the individual columns of the matrix. Thus, the PCA in this study takes the following form:

9 $$X=TP^{\prime} +r,$$

where *X* is a matrix containing the time series (*X* has 200 rows in this process, and the columns represent imagery epochs in each dataset), *T* is a matrix containing the scores, *P* is the loading (this matrix is transposed), and *r* represents the residuals, which are the unexplained portions of the time series in this model. We note that *T* and *P* are determined using the least squares method and that each PC contains one score and loading value, whereas PC1, PC2 and PC3 for each dataset have the highest possible variances.

### Data availability

All of the analysed SBAS and water level time series data collected during this study are included in this published article and the Supplementary Information files.

## Electronic supplementary material

Supplementary materials

Dataset 1
