# Supplementary material for: Spatiotemporal deformation patterns of the Lake Urmia Causeway as characterized by multisensor InSAR analysis
Source: Sci Rep. 2018 Apr 3;8:5357. doi: 10.1038/s41598-018-23650-6 (PMC5882932; doi:10.1038/s41598-018-23650-6)
Supplement: Supplementary file 1 — Supplementary materials [file 41598_2018_23650_MOESM1_ESM.doc]

**Supplementary materials for:**

Spatiotemporal deformation patterns of the Lake Urmia Causeway as characterized by multisensor InSAR analysis

Sadra Karimzadeh^1,2^*, Masashi Matsuoka^1^ & Fumitaka Ogushi^1^

^1^ Department of Architecture and Building engineering, Tokyo Institute of Technology, Japan

^2^ Department of GIS and Remote Sensing, University of Tabriz, Iran

*Corresponding author Tel: (+81)9072308182

Email: sadra.karimzadeh@gmail.com

* supplementary.xlsx also contains PCA loadings, PCA scores, water level data and all InSAR time series used in this study.

**Supplementary Table 1.** Detailed SAR information used in this study. * represents the reference scene.

| Label | Satellite | Band | Date | Baseline relative to reference image (m) | Polarization |
| --- | --- | --- | --- | --- | --- |
| 0 | ENVISAT | C-band | 2004.07.07 | 160.341 | VV |
| 2 | ENVISAT | C-band | 2004.12.29 | 480.162 | VV |
| 3 | ENVISAT | C-band | 2005.02.02 | -294.726 | VV |
| 4 | ENVISAT | C-band | 2005.05.18 | -332.21 | VV |
| 5 | ENVISAT | C-band | 2005.07.27 | -82.1946 | VV |
| 6 | ENVISAT | C-band | 2005.08.31 | -64.7057 | VV |
| 7 | ENVISAT | C-band | 2006.03.29 | -335.694 | VV |
| 8* | ENVISAT | C-band | 2006.06.07 | 0* | VV |
| 9 | ENVISAT | C-band | 2007.01.03 | 215.86 | VV |
| 10 | ENVISAT | C-band | 2007.05.23 | -211.395 | VV |
| 11 | ENVISAT | C-band | 2008.04.02 | 74.4041 | VV |
| 0 | ALOS-1 | L-band | 2006.12.26 | -1066.03 | HH |
| 1 | ALOS-1 | L-band | 2007.09.28 | 1237.33 | HH/HV |
| 2 | ALOS-1 | L-band | 2007.11.13 | 1853.23 | HH |
| 3 | ALOS-1 | L-band | 2007.12.29 | 1582.3 | HH |
| 4* | ALOS-1 | L-band | 2008.06.30 | 0* | HH/HV |
| 5 | ALOS-1 | L-band | 2008.12.31 | -1308.75 | HH |
| 6 | ALOS-1 | L-band | 2009.10.03 | 473.373 | HH/HV |
| 7 | ALOS-1 | L-band | 2010.01.03 | 987.656 | HH |
| 8 | ALOS-1 | L-band | 2010.02.18 | 1560.92 | HH |
| 9 | ALOS-1 | L-band | 2010.04.05 | 1690.95 | HH |
| 10 | ALOS-1 | L-band | 2010.05.21 | 1875.32 | HH/HV |
| 11 | ALOS-1 | L-band | 2010.07.06 | 1965.84 | HH/HV |
| 12 | ALOS-1 | L-band | 2010.10.06 | 2515.42 | HH/HV |
| 0 | TerraSAR-X | X-band | 2012.05.20 | -67.6469 | HH |
| 1 | TerraSAR-X | X-band | 2012.06.11 | -201.139 | HH |
| 2 | TerraSAR-X | X-band | 2012.07.03 | -83.498 | HH |
| 3 | TerraSAR-X | X-band | 2012.08.05 | -28.8052 | HH |
| 4 | TerraSAR-X | X-band | 2012.09.29 | -12.3271 | HH |
| 5* | TerraSAR-X | X-band | 2012.11.01 | 0* | HH |
| 6 | TerraSAR-X | X-band | 2012.12.15 | 108.227 | HH |
| 7 | TerraSAR-X | X-band | 2013.01.06 | -134.102 | HH |
| 8 | TerraSAR-X | X-band | 2013.01.28 | -116.674 | HH |
| 9 | TerraSAR-X | X-band | 2013.04.04 | -204.566 | HH |
| 10 | TerraSAR-X | X-band | 2013.05.18 | -175.475 | HH |
| 11 | TerraSAR-X | X-band | 2013.07.01 | -18.2047 | HH |
| 12 | TerraSAR-X | X-band | 2013.07.23 | -63.2604 | HH |
| 13 | TerraSAR-X | X-band | 2013.08.25 | -381.24 | HH |
| 14 | TerraSAR-X | X-band | 2013.10.08 | -120.567 | HH |
| 0 | Sentinel-1 | C-band | 2015.11.11 | -37.6333 | VV/VH |
| 1 | Sentinel-1 | C-band | 2015.12.05 | 39.707 | VV/VH |
| 2 | Sentinel-1 | C-band | 2015.12.29 | 94.7522 | VV/VH |
| 3 | Sentinel-1 | C-band | 2016.01.22 | 44.7473 | VV/VH |
| 4 | Sentinel-1 | C-band | 2016.02.15 | -29.1692 | VV/VH |
| 5 | Sentinel-1 | C-band | 2016.03.10 | 11.587 | VV/VH |
| 6 | Sentinel-1 | C-band | 2016.04.03 | 22.3689 | VV/VH |
| 7 | Sentinel-1 | C-band | 2016.04.27 | -15.6678 | VV/VH |
| 8 | Sentinel-1 | C-band | 2016.05.21 | 37.7035 | VV/VH |
| 9 | Sentinel-1 | C-band | 2016.06.14 | 2.97016 | VV/VH |
| 10 | Sentinel-1 | C-band | 2016.07.08 | 25.6155 | VV/VH |
| 11 | Sentinel-1 | C-band | 2016.08.01 | 55.5624 | VV/VH |
| 12 | Sentinel-1 | C-band | 2016.09.18 | 32.2466 | VV/VH |
| 13 | Sentinel-1 | C-band | 2016.11.05 | 55.1289 | VV/VH |
| 14 | Sentinel-1 | C-band | 2016.11.29 | 90.0376 | VV/VH |
| 15 | Sentinel-1 | C-band | 2016.12.23 | 17.2635 | VV/VH |
| 16 | Sentinel-1 | C-band | 2017.01.16 | 37.5671 | VV/VH |
| 17 | Sentinel-1 | C-band | 2017.02.21 | -25.2961 | VV/VH |
| 18 | Sentinel-1 | C-band | 2017.03.05 | -104.271 | VV/VH |
| 19 | Sentinel-1 | C-band | 2017.03.17 | -5.01973 | VV/VH |
| 20 | Sentinel-1 | C-band | 2017.03.29 | 50.1012 | VV/VH |
| 21 | Sentinel-1 | C-band | 2017.04.10 | 58.3419 | VV/VH |
| 22* | Sentinel-1 | C-band | 2017.05.04 | 0* | VV/VH |
| 23 | Sentinel-1 | C-band | 2017.05.16 | 24.9512 | VV/VH |
| 24 | Sentinel-1 | C-band | 2017.05.28 | -41.4881 | VV/VH |
| 25 | Sentinel-1 | C-band | 2017.06.09 | 79.4127 | VV/VH |
| 26 | Sentinel-1 | C-band | 2017.06.21 | 38.952 | VV/VH |
| 27 | Sentinel-1 | C-band | 2017.07.03 | -41.0752 | VV/VH |

**Supplementary Table 2.** Results of the ADF test for the first three principle components.

| PC | ADF assumption | P-value | Sensor | Stationary (if P-value< significance level) | Significance level |
| --- | --- | --- | --- | --- | --- |
| 1 | No constant | 24.8% | Envisat | False | 5.0% |
|  |  | 70.6% | ALOS-1 | False |  |
|  |  | 90.5% | TSX | False |  |
|  |  | 68.8% | Sentinel-1 | False |  |
|  | Constant only | 0.1% | Envisat | True | 5.0% |
|  |  | 3.2% | ALOS-1 | True |  |
|  |  | 2.6% | TSX | True |  |
|  |  | 0.4% | Sentinel-1 | True |  |
|  | Constant + Trend | 0.0% | Envisat | True | 5.0% |
|  |  | 3.5% | ALOS-1 | True |  |
|  |  | 0.1% | TSX | True |  |
|  |  | 0.0% | Sentinel-1 | True |  |
| 2 | No constant | 68.8% | Envisat | False | 5.0% |
|  |  | 13.3% | ALOS-1 | False |  |
|  |  | 11.1% | TSX | False |  |
|  |  | 8.7% | Sentinel-1 | False |  |
|  | Constant only | 0.5% | Envisat | True | 5.0% |
|  |  | 0.9% | ALOS-1 | True |  |
|  |  | 64.2% | TSX | False |  |
|  |  | 3.6% | Sentinel-1 | True |  |
|  | Constant + Trend | 0.0% | Envisat | True | 5.0% |
|  |  | 17% | ALOS-1 | False |  |
|  |  | 10% | TSX | False |  |
|  |  | 5% | Sentinel-1 | True |  |
| 3 | No constant | 0.1% | Envisat | True | 5.0% |
|  |  | 7% | ALOS-1 | False |  |
|  |  | 0.1% | TSX | True |  |
|  |  | 0.1% | Sentinel-1 | True |  |
|  | Constant only | 0.1% | Envisat | True | 5.0% |
|  |  | 9% | ALOS-1 | False |  |
|  |  | 4.8% | TSX | True |  |
|  |  | 1.4% | Sentinel-1 | True |  |
|  | Constant + Trend | 0.1% | Envisat | True | 5.0% |
|  |  | 8% | ALOS-1 | False |  |
|  |  | 0.1% | TSX | True |  |
|  |  | 0.0% | Sentinel-1 | True |  |


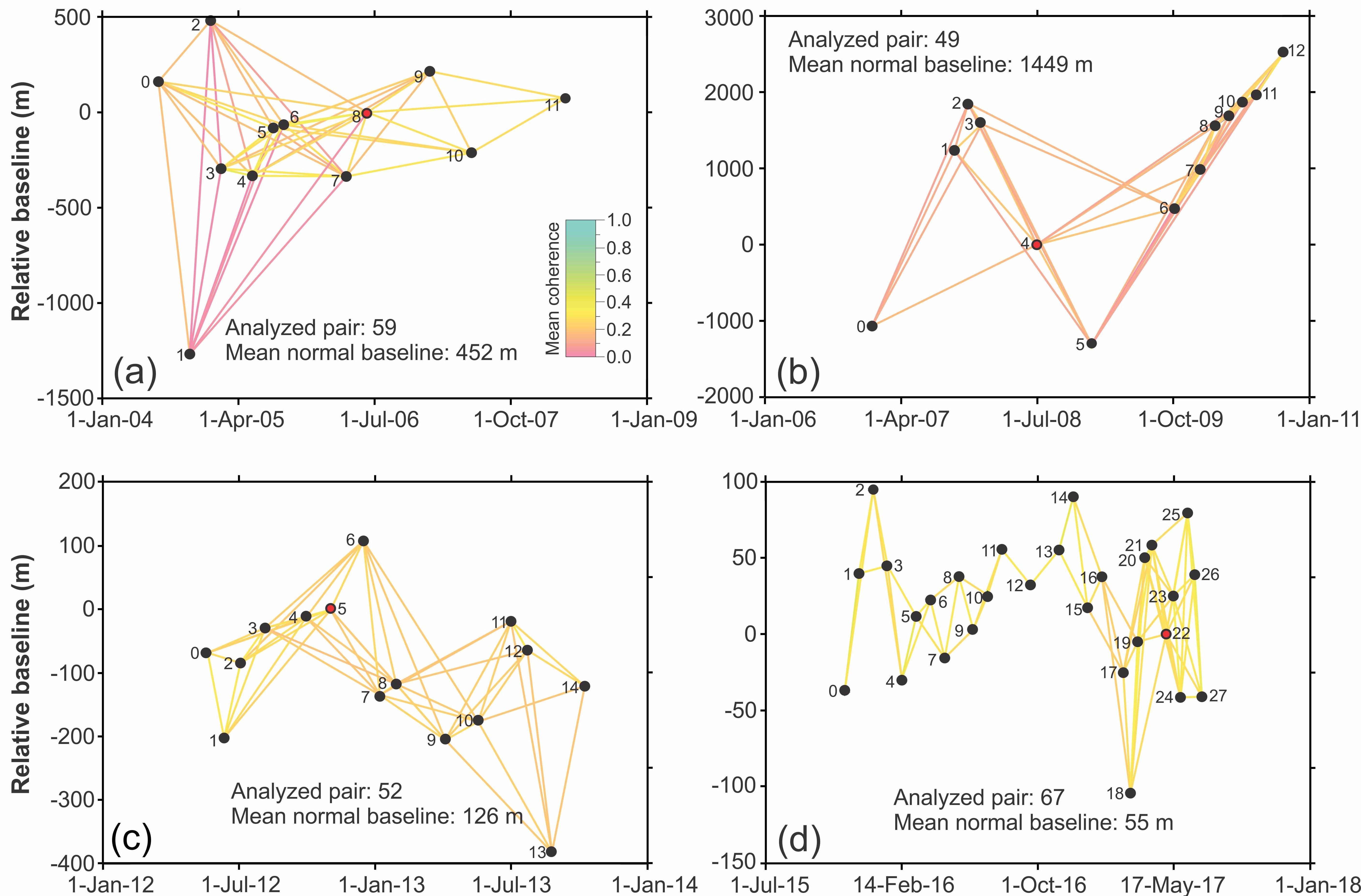


**Supplementary Fig. 1.** SBAS coherence network of (a) Envisat, (b) ALOS-1, (c) TSX and (d) Sentinel-1. Coloured lines indicate mean coherence values, and red circles indicate reference images in each network.


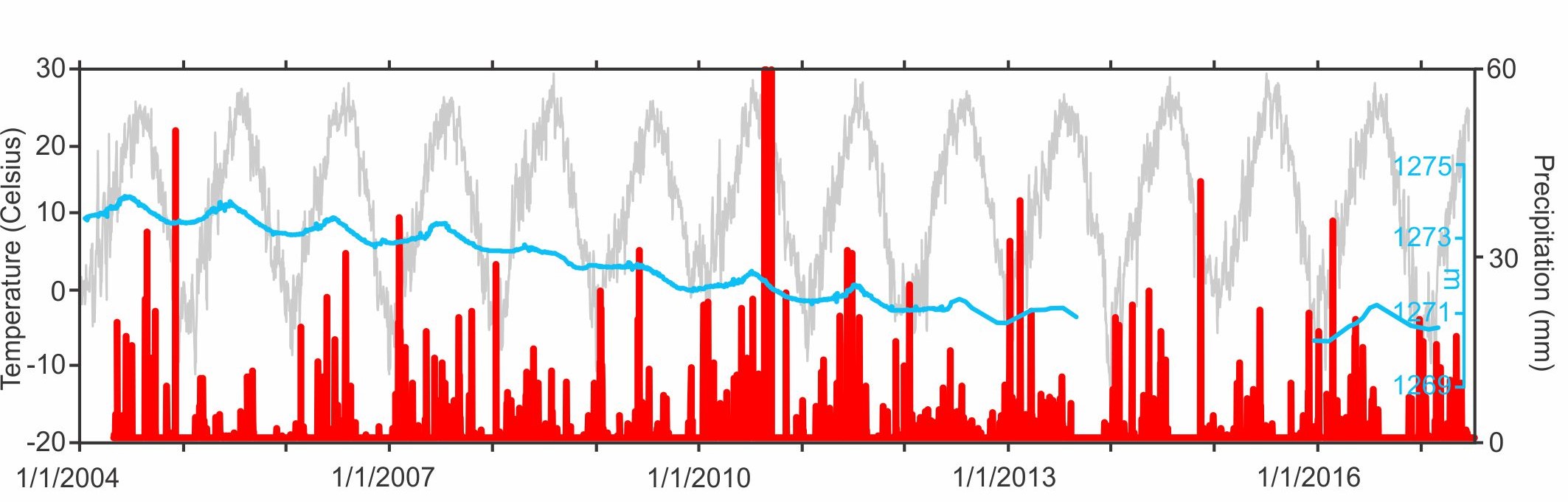


**Supplementary Fig.2.** Progressive water level (blue) decline together with temperature (grey) and precipitation (red) fluctuations of the Lake Urmia. Note, water level information from 9 October 2013 to 10 November 2015 is lacking.


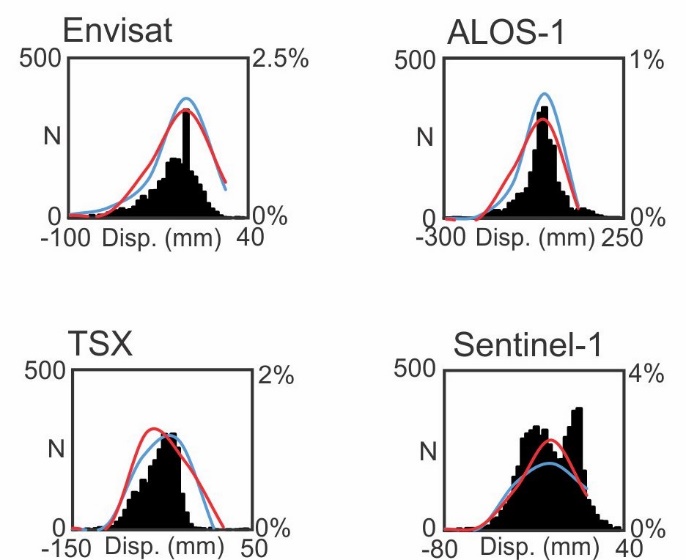


**Supplementary Fig. 3.** Histograms of the selected 200 time series together with Gaussian (red) and Kernel Density (blue) estimations.


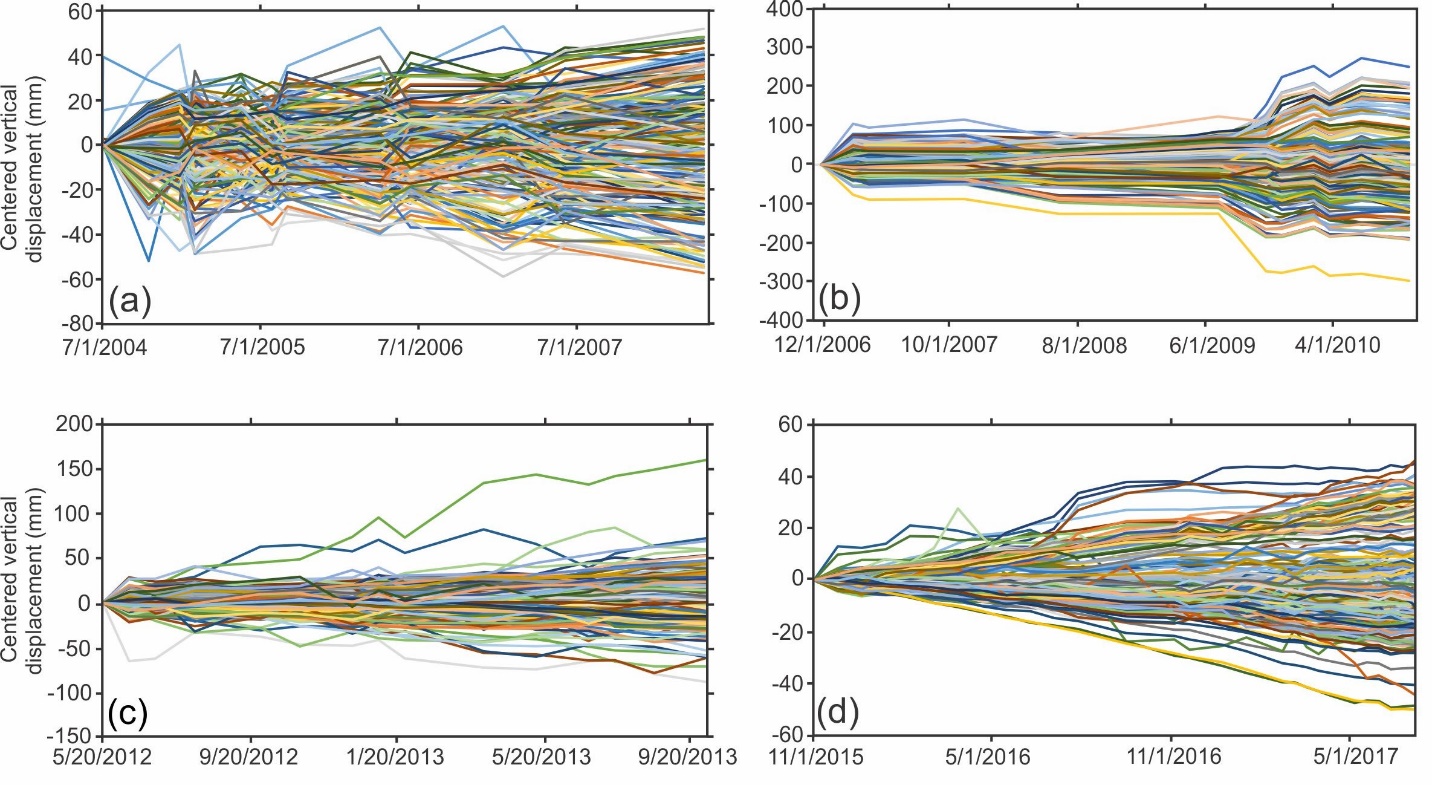


**Supplementary Fig. 4.** Centred time series of 200 samples by reducing the mean value from the samples. (a) Envisat, (b) ALOS-1, (c) TSX and (d) Sentinel-1.
